# Optimal Installation Locations for Automated External Defibrillators in Taipei 7-Eleven Stores: Using GIS and a Genetic Algorithm with a New Stirring Operator

**DOI:** 10.1155/2014/241435

**Published:** 2014-06-17

**Authors:** Chung-Yuan Huang, Tzai-Hung Wen

**Affiliations:** ^1^Department of Computer Science and Information Engineering, School of Electrical and Computer Engineering, College of Engineering, Chang Gung University, 259 Wen Hwa 1st Road, Taoyuan 333, Taiwan; ^2^Department of Geography, National Taiwan University, No. 1, Section 4, Roosevelt Road, Taipei 10617, Taiwan; ^3^Infectious Disease Research and Education Center (DOH-NTU), No. 17, Hsu-Chu Road, Taipei 100, Taiwan

## Abstract

Immediate treatment with an automated external defibrillator (AED) increases out-of-hospital cardiac arrest (OHCA) patient survival potential. While considerable attention has been given to determining optimal public AED locations, spatial and temporal factors such as time of day and distance from emergency medical services (EMSs) are understudied. Here we describe a geocomputational genetic algorithm with a new stirring operator (GANSO) that considers spatial and temporal cardiac arrest occurrence factors when assessing the feasibility of using Taipei 7-Eleven stores as installation locations for AEDs. Our model is based on two AED conveyance modes, walking/running and driving, involving service distances of 100 and 300 meters, respectively. Our results suggest different AED allocation strategies involving convenience stores in urban settings. In commercial areas, such installations can compensate for temporal gaps in EMS locations when responding to nighttime OHCA incidents. In residential areas, store installations can compensate for long distances from fire stations, where AEDs are currently held in Taipei.

## 1. Introduction

The American Heart Association defines out-of-hospital cardiac arrest (OHCA) as the cessation of mechanical cardiac activity outside of a medical care setting as confirmed by the absence of circulation [1, 2]. Survival is strongly correlated with time between OHCA occurrence and first defibrillation, with 4 minutes or less considered as optimum for survival [3, 4]. Survival potential declines by 7–10% for each minute that treatment is delayed [5]. For 80% of OHCA patients whose events occur at home and 60% whose events occur in the presence of another individual [6], survival is heavily dependent on the dispatch point of the nearest ambulance or emergency medical service (EMS) team [7, 8]. Accordingly, EMS timeliness is a top priority for emergency medicine researchers [9]. While cardiopulmonary resuscitation (CPR) performed while waiting for other forms of treatment increases OHCA survival by approximately 8% [10], CPR requires voluntary participation in a 2-3 hour training session. The installation of automated external defibrillators (AEDs) in public locations is therefore considered the most effective means of reducing the time from OHCA onset to first defibrillation [11–13]. Early defibrillation programs in Taipei began in June of 2000 [14], with the immediate goal of making AEDs available in public locations such as airports, fire stations, university campuses, and shopping malls.

Despite the research attention given to spatial analytical methods and patterns in the design of medical services [15, 16], few efforts have been made to thoroughly study the spatial and temporal aspects of OHCA incidents and EMS availability when determining optimal locations for AED installations [12, 17, 18]. In terms of temporal variation, lower cardiac arrest rates at night are likely due to less human activity in public areas, reducing the probability of OHCA discovery and increasing treatment delays. In light of evidence indicating variance in temporal OHCA frequency [19], defibrillator installation planners must identify areas with higher rates of cardiac arrest to increase the potential for bystanders to provide emergency treatment prior to the arrival of trained emergency personnel. In terms of spatial variation, optimal AED installation location can compensate for geographic obstacles to deliver timely EMS treatment [7]. Defibrillators have been installed in many cities at fixed locations such as parks, shopping malls, bus stops, and airports [17]. Despite the easy identification of AEDs installed at fixed locations, much higher concentrations are found in high-activity areas, leaving low-activity areas underserved. Fixed AED locations must therefore be supplemented by mobile AEDs in police cars and other emergency vehicles [20]. However, since public safety personnel are often occupied by other emergencies, fixed location for AEDs is the favored approach.

In this paper, we describe a* geocomputational genetic algorithm with a new stirring operator* (GANSO) that considers spatial and temporal cardiac arrest occurrence factors when assessing the feasibility of using Taipei 7-Eleven stores as installation locations for AEDs. Taipei has the highest density of 24-hour convenience stores in the world, nearly 2,000 serving the needs of over two million residents. The country's public health officials are examining the feasibility of installing AEDs in many of these stores to compensate for the shortage of EMSs in locations away from urban centers and in commercial neighborhoods with less activity during nighttime hours. Given the prohibitive cost of installing an AED in every 7-Eleven store, an appropriate subset of locations providing the greatest coverage must be identified [21, 22]. This task requires consideration of spatial and temporal factors including OHCA onset time, location, frequency, distance between potential AED locations, and distance to EMS facilities (usually fire stations). It can be formulated as either a location-allocation optimization problem [21, 22] or a weighted set-covering problem (i.e., a nondeterministic polynomial time complete [NP-complete] problem) [23]. Computational complexity can be reduced by using a weighted set-covering genetic algorithm (GA) (in location-allocation optimization problems involving EMSs [24, 25], GAs offer high utility value in scenarios involving geographic information systems (GISs) [26]—for example, when comparing response times for evaluating EMS and ambulance allocation [27]; GAs have also been used to maximize coverage for fire stations, post offices, and banks [22]; GA performance and convergence are closely associated with appropriate methods for encoding candidate solutions, objective functions, selection and reproduction operators, parameter settings, and specific success criteria [25, 28]) to obtain a set of approximation solutions [29, 30].

## 2. Materials and Methods

**Figure 1:**
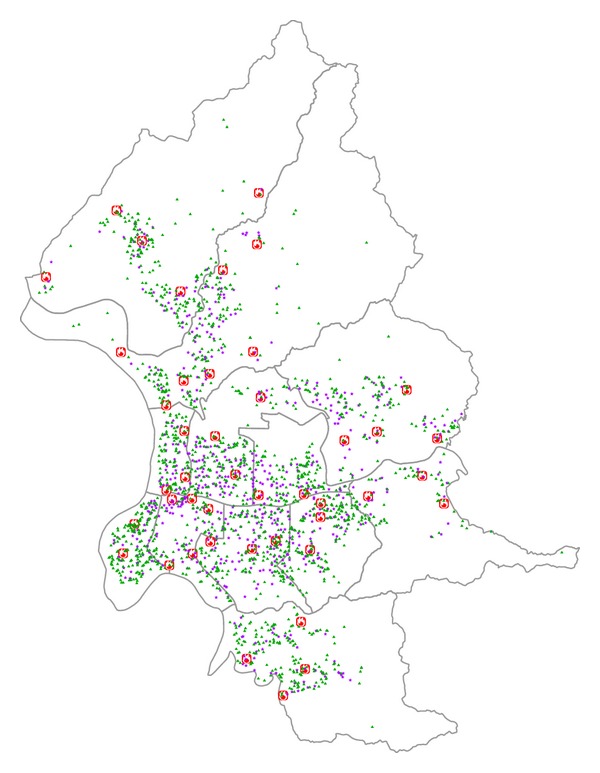
Geographic distribution of 1,625 OHCA cases, 677 7-Eleven stores, and 44 fire stations in Taipei in 2010. Each green dot denotes one OHCA case, each purple dot one 7-Eleven store, and each red icon one fire station.

Three categories of data were used in this study: OHCA information, 7-Eleven locations, and fire station locations. OHCA data are from the Emergency Medical Service Registry System of the Taipei City Government. Filter criteria for the OHCA cases include nontrauma events between January and December of 2010 involving patients over 18 years of age who were treated by emergency medical services personnel. The registry system was established by the Taipei City Department of Health. Data were compiled by the City's Fire Department and Taipei area hospitals. OHCA data include time and date of onset, location, ambulance/EMS vehicle response time, and type of cardiovascular life support used (advanced, basic, or a combination). Convenience store and fire station data were collected from the 7-Eleven Chain Store Corporation and Taipei City Fire Department. Geographic distributions of OHCA patients, convenience stores, and fire stations are shown in Figure 1.

Our proposed framework consists of two stages: (a) determining the spatial and temporal weights of OHCA incidents and covering sets for individual convenience stores and (b) using GANSO with first-stage findings to solve AED location-allocation optimization problems entailing spatial and temporal variation. We will describe two scenarios for determining AED conveyance from convenience stores to cardiac arrest victims, with service distance set at either 100 or 300 meters. At 100 meters, the primary conveyance mode is running at an average speed of 80–100 meters per minute. This allows for defibrillation within the four-minute time span considered optimal for saving the lives of OHCA victims. When the service distance is set at 300 meters, a vehicle moving at an average speed of 320–400 meters per minute is required to stay below the four-minute target.

### 2.1. Temporally and Spatially Weighted Models

We assumed a total of *M* OHCA patients (labeled 1 to *M*), *N* 7-Eleven stores (labeled 1 to *N*), and *L* fire stations (labeled 1 to *L*) in Taipei in 2010. Each OHCA patient is denoted as an object variable *o*_*i*_  (*i* ∈ {1,2,…, *M*}) belonging to a patient set *O* = {*o*_1_, *o*_2_,…, *o*_*M*_} consisting of all *M* OHCA patients. Each 7-Eleven store is denoted as an object variable *c*_*j*_  (*j* ∈ {1,2,…, *N*}) belonging to a store set *C* = {*c*_1_, *c*_2_,…, *c*_*N*_} consisting of all *N* 7-Eleven stores. Subscript *j* on store object *c* is also used to denote the serial number of 7-Eleven stores (i.e.,  *c*_*j*_.*serial*_*no* = *j*). Each fire station is denoted by an object variable *f*_*k*_  (*k* ∈ {1, 2,…, *L*}) belonging to a set *F* = {*f*_1_, *f*_2_,…, *f*_*L*,_} consisting of all *L* fire stations.

Each OHCA patient object *o*_*i*_ had three major member attributes (sometimes referred to as “properties”):* event_time*,* event_date,* and* loc*. The* event_time* attribute denotes the onset time of an OHCA event using the format* hh*:*mm*:*ss* (24-hour clock—00:00:00 to 23:59:59),* event_date* denotes the onset date of an OHCA event using the format* yyyy*.*mm*.*dd*., and* loc* denotes the onset location of an OHCA event using an (*x*, *y*) GPS geographic position format. Next, we developed two weighting schemes to capture spatial and temporal variation for AED location selection: (a) occurrence frequency and time of cardiac arrest in a given location for temporal variation and (b) distance between EMS facility and convenience store for spatial variation. We assumed that convenience store AEDs compensate for lost time when they are located far from the closest EMS facilities.

For the TWM parameters, time of cardiac arrest event was categorized in terms of month and time period. An attempt was made to determine the monthly temporal weight weight(*o*_*i*_, *mon*) of each OHCA patient. We considered situations in which the OHCA event onset month* equaled a mon parameter value *(i.e., *mon* *t* *h*(*o*_*i*_.*event*_*date*) = *mon*), with a function weight(*o*_*i*_, *mon*) return of 1.0 (nighttime) or 0.5 (daytime) according to subfunction weight(*o*_*i*_), depending on what time of day the event occurred. Otherwise, the weight(*o*_*i*_, *mon*) function returns an interpolated value indicating the sum of the weighted OHCA, standardized in terms of inverse distance weighting:

(1) weight(oi,mon)={weight(oi)month(oi.event_date)=mon∑month(oj.event_date)=mon weight(oj)×distance_weight(oi,oj)∑k≠i∧month(ok.event_date)=mondistance_weight(oi,ok)otherwise,

where

(2) $$\begin{array}{c} \text{weight}\left(o_{i}\right)=\{\begin{array}{cc} 0.5 & \text{if}\;\text{time}\_\text{period}\left(o_{i}.event\_time\right)\;\\ & ="\text{daytime}"\\ 1.0 & \text{if}\;\text{time}\_\text{period}\left(o_{i}.event\_time\right)\\ & ="\text{nighttime}", \end{array}\\ \text{distance}\_\text{weight}\left(o_{i},o_{j}\right)=\frac{1}{\text{distance}{\left(o_{i}.loc,o_{j}.loc\right)}^{2}}. \end{array}$$

Three user-defined functions were used to determine the monthly temporal weight of an OHCA patient: month (*date*) returns the month from a passed date parameter. The time_period (*time*) returns one of two period strings from a passed time parameter—08:00 to 17:00 representing “daytime” and all other values “nighttime.” The distance (*loc*_*i*_, *loc*_*j*_) returns a Euclidean distance between two passed position parameters of a plane with Cartesian coordinates on a map. The functions of time_period (*time*) and distance (*loc*_*i*_, *loc*_*j*_) are defined as follows:

(3) time_period(time) ={"daytime"08:00:00≤time≤17:00:00"nighttime"otherwise,distance(loci,locj) =(loci.x−locj.x)2+(loci.y−locj.y)2.

Next, we computed an OHCA temporal weight by combining 12 monthly weight values (weight (*o*_*i*_, 1), weight (*o*_*i*_, 2),…, weight (*o*_*i*_, 12)). A coefficient of variation (COV), defined as a ratio of the mean mean (*o*_*i*_) to standard deviation *sd*⁡ (*o*_*i*_), served as the OHCA weight—that is,

(4) $$\begin{array}{c} \text{temporal}\_\text{wieght}\left(o_{i}\right)=\frac{\text{mean}\left(o_{i}\right)}{\mathrm{sd}\left(o_{i}\right)} \end{array}$$

with mean (*o*_*i*_) and *sd*⁡ (*o*_*i*_) computed as

(5) $$\begin{array}{c} \text{mean}\left(o_{i}\right)=\frac{\sum_{m=1}^{12}\text{weight}\left(o_{i},m\right)}{12},\\ \mathrm{sd}\left(o_{i}\right)=\sqrt{\frac{\sum_{m=1}^{12}{\left(\text{weight}(o_{i},m)-\text{mean}\left(o_{i}\right)\right)}^{2}}{12}.} \end{array}$$

In the SWM, a higher weight indicates a longer distance to the nearest fire station. Spatial weight was assumed as the minimum square Manhattan distance to each fire station (*f*_*k*_):

(6) spatial_weight(oi)  =min⁡k  Manhattan_distance (oi.loc,fk.loc)2.

Due to the grid-like characteristic of urban street networks, we used Manhattan distances to compute distances from fire stations to OHCA victims expressed as

(7) Manhattan_distance(loci,locj) =|loci.x−locj.x|+|loci.y−locj.y|.

### 2.2. Genetic Algorithm with a New Stirring Operator (GANSO)

We formulated the AED location-allocation optimization problem as a choice within a limited *K*-sized subset of convenience stores, using GANSO to determine an optimum subset based on the temporal or spatial characteristics of OHCA victims. As shown in Pseudocode 1, our proposed GANSO (GANSO was implemented as a Python program, which supports future simulation experiments and possible extensions; all input/output data from our application were managed and processed using Quantum GIS (QGIS), an open source (general public license) GIS; for source code and input data for OHCA information, 7-Eleven locations, and fire station locations, contact the corresponding author) consists of nine steps.

**Pseudocode 1:**
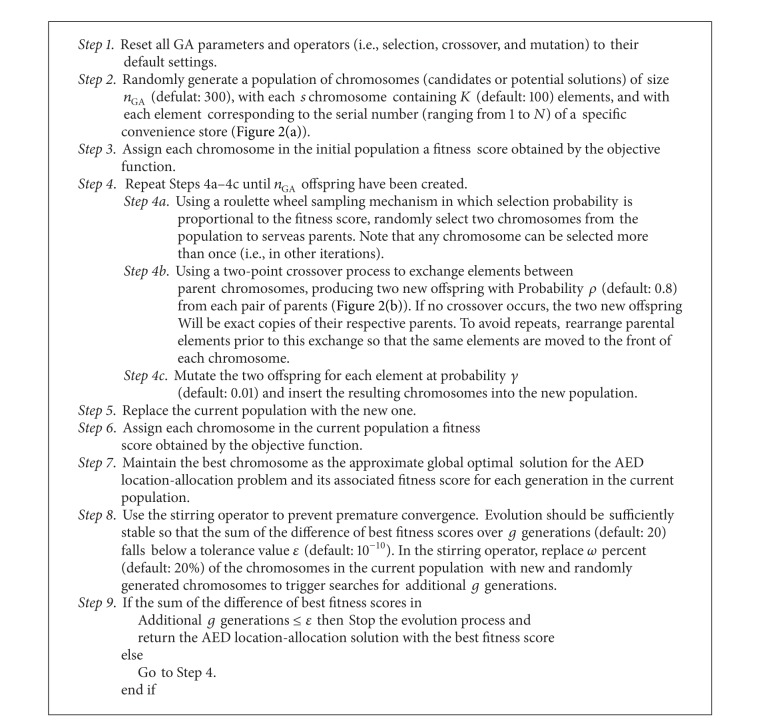


In Step 2, population size *n*_GA_ depends on both the individual chromosome length and fitness function details. For this project, a population size between 200 and 400 chromosomes generated good solutions for the AED location-allocation problem after testing trial runs of up to 1,000 chromosomes. The evolutionary process from Steps 4 to 10 represents an equilibrium between “exploring” the entire search space (2^10*K*^) and “exploiting” the current best solution to search for an optimal or near-optimal AED location-allocation solution. Although crossover rate *ρ* in Step 4b is generally applied with a high probability (0.7 to 0.9), excessive use of the crossover operation can disrupt the balance of this equilibrium and create excessive diversity to cause the process to drift from a fit structure. Inadequate diversity can also result in premature convergence and a locally optimum rather than globally fit structure. A balance between exploration and exploitation increases the probability of achieving a global optimum solution, but the *γ* mutation rate in Step 4c is normally set at a very low probability (e.g., 0.01). A higher mutation rate reproduces an excessive number of random populations, which can also impair the evolutionary mechanism [26]. In GANSO, crossover rate *ρ* and mutation rate *γ* are set at 0.8 and 0.01, respectively, to achieve an optimum outcome.

To evaluate the fitness score of each chromosome during the GANSO evolution process, each *K*-element chromosome is transformed into a one-dimensional binary array *X* of length *N* corresponding to index *X*[*j*]  (*j* ∈ {1,2,…, *N*}) of convenience store candidates (*X*[*j*] = 1 denoting the chosen index, 0 otherwise) for use as a parameter for the following location-allocation optimization problem fitness function:

(8) fitness(X)=maximize ∑i=1MW[i,]∘X  subject  to ∑j=1NX[j]=K,transform(chromosome  s)=  declare  variable  X  as  an  array[1…N]  of binary integer  for each index in range(1,N)  do loop    X[index]⟵0  for  each  index  in  range(1,K)  do  loop    sotre_serial_no⟵Integer(s[indexs])    X[store_serial_no]⟵1  return  X,

where *W* denotes an *M*-row by *N*-column weighting matrix determined by the temporal or spatial characteristics of all OHCA patients in question and *W*[*i*, ] refers to the *i*th row of the constant weighting matrix *W*, with *W*[*i*, *j*] representing the weight of the *i*th OHCA patient covered by the *j*th 7-Eleven store. The ∘ operator is defined as the maximum value among chosen elements:

(9) $$\begin{array}{c} W_{i}\circ X=\underset{j}{\max}\left(W\left[i,j\right]\cdot X\left[j\right]\right). \end{array}$$

The covering set consists of all 7-Eleven stores in Taipei: for each store, the coverable object is one OHCA incident. Coverable OHCAs are assumed for each *c*_*j*_ cutoff by a constant distance_threshold as follows:

(10) oi∈cj⟷Manhattan_distance(oi.loc,cj.loc)     ≤distance_threshold.

Correspondingly, the *W* constant weighting matrix is defined as

(11) $$\begin{array}{c} W\left[i,j\right]=\{\begin{array}{cc} \text{model}\_\text{weight}\left(o_{i}\right) & o_{i}\in c_{j}\\ & ⟷\text{Manhattan}\_\text{distance}\\ & \;\;\left(o_{i}.loc,c_{j}.loc\right)\\ & \;\;\leq distance\_threshold\\ 0 & \text{otherwise}, \end{array} \end{array}$$

where

(12) model_weight(oi) ={temporal_weight(oi)if  the  adopted  weighting  model  is  "TWM"spatial_weight(oi)if  the  adopted  weighting  model  is  "SWM"  ,distance_threshold ={100when  the  maximal  service  distance  was  set  at  100  meters300when  the  maximal  service  distance  was  set  at  300  meters.

**Figure 3:**
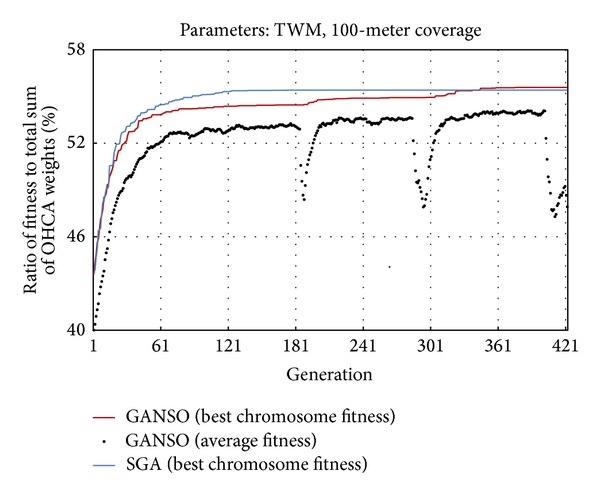
Comparison of GANSO and SGA performance over 421 generations.

Figure 3 presents a summary of results from a single TWM experiment using 100 meters as the service distance. As shown, our proposed GANSO (Figure 3, red curve) outperformed simple genetic algorithm (SGA) (blue curve). The *x*-axis denotes the evolutionary generation number and the *y*-axis the ratio of the fitness score (the sum of weights for OHCA cases covered by 100 7-Eleven stores) to the total sum of OHCA weights. Experiment results indicate that the new stirring operator was effective in addressing evolution time and performance issues. While GANSO performed poorly during the first few generations, its overall performance was satisfactory: by stirring the population, its best fitness values gradually increased over 180–400 generations, yielding better solutions for AED location selection.

## 3. Results and Discussion

**Figure 4:**
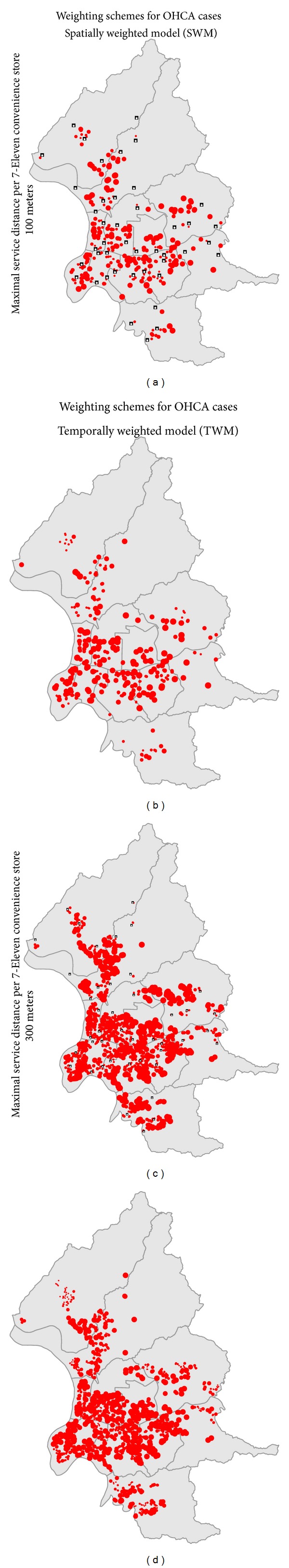
Spatial and temporal weight distributions of OHCA cases. (a) SWM, 100-meter coverage; (b) TWM, 100-meter coverage; (c) SWM, 300-meter coverage; and (d) TWM, 300-meter coverage. Red dots denote OHCA case weights.

At a service distance of 100 meters, 19.9% (323 cases) of the 1,625 cardiac arrest victims were within adequate coverage zones of 38.7% (262) of the 677 7-Eleven stores operating in Taipei. When the distance was increased to 300 meters, 78.2% (1,271 cases) were within the adequate coverage zones of 92% (623) of those stores. Spatial and temporal weight distribution patterns for the OHCA cases are shown in Figure 4; larger red dots denote higher SWM and TWM weights. Note that TWM-associated OHCA weights were concentrated in west Taipei, which has a higher population density. In comparison, OHCA weights associated with SWM cases were uniformly distributed throughout the city.

**Figure 5:**
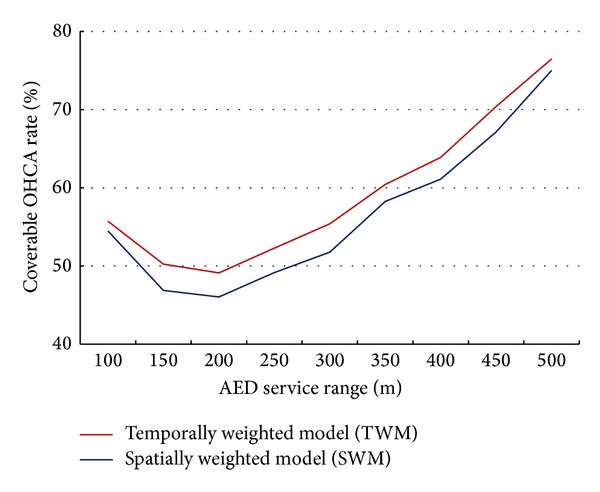
Comparison of coverable OHCA rates for different AED service ranges when the number of 7-Eleven convenience stores is limited to 100.

Optimal solutions are summarized in Table 1. When the number of stores was limited to 100 and the service distance was fixed at either 100 or 300 meters, coverage was higher for the TWM parameters—specifically, 180 OHCA patients (55.7%) compared to 176 (54.5%) for the SWM parameters. When the service distance was increased to 300 meters, 704 cases (55.4%) were covered by the TWM and 658 (51.8%) by the SWM parameters. Overall, the TWM and SWM coverage rates for 100 meters were higher than for 300 meters (55.7% versus 55.4% and 54.5% versus 51.8%, resp.).  Table 2 and Figure 5 present a detailed comparison of optimal solutions for different service ranges (from 100 to 500 meters in steps of 50 meters) and weighting schemes (TWM and SWM) when the number of stores earmarked for installing AEDs was limited to between 100 and 200.

**Figure 6:**
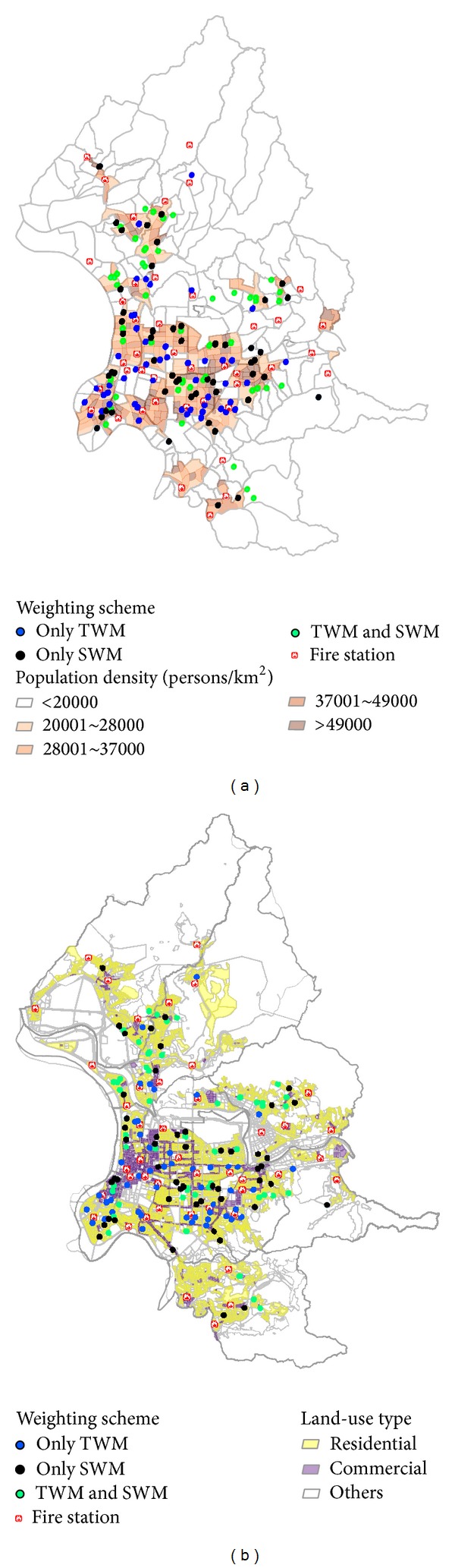
Spatial distributions of (a) population density and (b) land-use type with locations of 7-Eleven stores selected based on SWM and TWM parameters with a 100-meter service range. Data are from 2010 census. Each dot denotes one store. Blue dots indicate stores selected according to TWM parameters and green dots according to SWM parameters. Black dots represent stores identified by both models.

As shown in Figure 6(a), most of the TWM store locations were in high-density areas and most of the SWM locations in lower-density areas, perhaps due to the TWM preference for locations with higher nighttime OHCA frequencies, as denoted by the large number of blue dots in the densely populated southwest area of Taipei. The SWM parameters indicated a preference for locations farther away from fire stations, which explains why most of the green dots are in outlying neighborhoods (see also Figure 6(a) and Table 3(a)). The overlapping areas marked by black dots indicate high-priority communities for AED installations in convenience stores.

According to Figure 6(b), the TWM parameters favored stores in commercial areas (blue dots), while the SWM parameters favored stores in residential areas (green dots). A higher OHCA incidence rate in commercial areas suggests higher levels of human activity. Note that the SWM parameters identified a larger number of AED installation points due to the smaller number of fire stations in residential areas and their greater distances from identified convenience stores. Although the TWM parameters determined more commercial locations, the SWM-selected locations were more uniform in both high- and low-density areas (Tables 3(b) and 4).

The tendencies of both models to select convenience stores in densely populated areas based on spatial and temporal considerations are consistent with those reported by Malcom III et al. [18]. They are likely due to the positive correlation between OHCA incidence rate and population density—a link that also explains the greater likelihood of identifying stores for AED installation in commercial areas characterized by higher levels of human activity. Further, optimal locations identified by the TWM parameters were more likely to be located in commercial areas and those identified by the SWM parameters in residential areas. These results are consistent with those reported by Folke et al. [31]. In addition to emphasizing the importance of temporal variation, our results underscore the need for more AED locations in commercial areas. Conversely, since residential areas in Taipei tend to have less business activity and fewer and more widely spaced fire stations, the SWM tendency to identify stores in residential neighborhoods may be viewed as compensating for lower EMS efficiency.

Whereas the SWM parameters gave higher priority to locations far from fire stations, the TWM parameters prioritized areas of greater human/commercial activity and locations (more residential than commercial) with higher nighttime OHCA incidence rates. This overlap also emphasizes the importance of installing AEDs in public locations to compensate for shortcomings in EMS facility numbers and locations. Regardless of the model used, the most efficient AED installations in convenience stores or other public locations must consider transport time to cardiac arrest victims. Determining the effects of heavy foot or vehicle traffic is extremely difficult; therefore, AED installation decisions must reflect OHCA incident frequencies and average traffic volumes. Our findings suggest that shorter service distances should be emphasized in areas where OHCA rates are higher and that longer distances are appropriate in areas with low traffic volumes.

## 4. Conclusion

In this paper, we presented a novel framework for solving the NP-complete weighted set-covering problem of allocating AEDs in a subset of 7-Eleven stores in Taipei. To develop a GA with a new stirring operator, we modified a simple GA by adding new chromosomes to a stable evolution process to prevent premature convergence. Two spatially and temporally weighted models (SWM and TWM) were created to consider the spatial and temporal characteristics of convenience stores and OHCA incidents in order to validate the feasibility of using our proposed GANSO to solve AED location-allocation optimization problems. Experiment results indicate that the highest priority for installing AEDs in Taipei communities should be given to convenience stores located in high-density areas. In commercial areas, AEDs in convenience stores can help compensate for temporal gaps in EMSs for cardiac arrest victims in general and for nighttime OHCA cases in particular. In residential areas, AEDs in convenience stores can help compensate for spatial gaps in terms of EMS delivery.

## Figures and Tables

**Figure 2:**
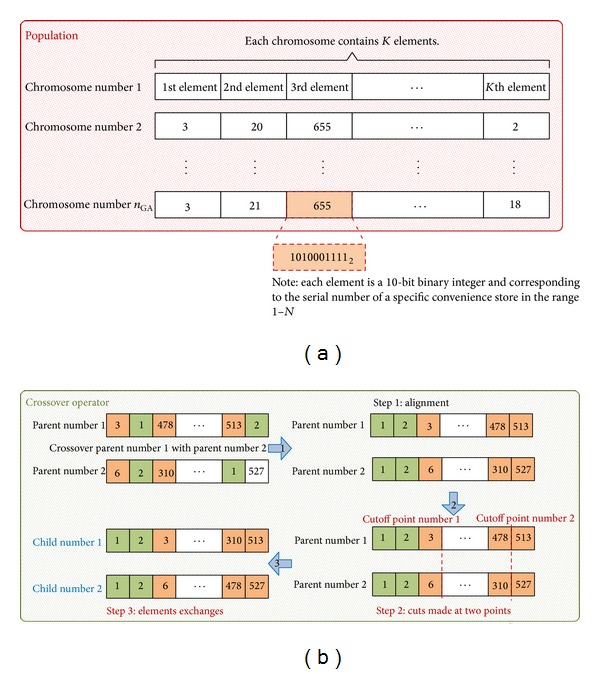
(a) Chromosome-encoding scheme. (b) Proposed GANSO crossover operator flow.

**Table 1:** Comparison of results for different parameter settings when the number of 7-Eleven convenience stores is limited to 100.

Parameter		Coverable OHCA cases	Coverable OHCA weights
AED service range	Weighting scheme		
100 meters	TWM	180 (55.73%)	56.23%
	SWM	176 (54.49%)	71.63%

300 meters	TWM	704 (55.39%)	53.88%
	SWM	658 (51.77%)	66.25%

**Table 2:** Comparison of results for different AED service ranges when the number of 7-Eleven convenience stores is limited to 100, 150, or 200.

Parameter				100 stores for AED installation		150 stores for AED installation		200 stores for AED installation	
AED service range	7-Eleven stores (*N* = 677)	OHCA (*M* = 1625)	Weighting scheme	Coverable OHCA cases	Coverable OHCA weights	Coverable OHCA cases	Coverable OHCA weights	Coverable OHCA cases	Coverable OHCA weights
100 meters	262 (38.70%)	323 (19.88%)	TWM	180 (55.73%)	56.23%	230 (71.21%)	73.04%	280 (86.69%)	88.40%
			SWM	176 (54.49%)	71.63%	229 (70.90%)	87.85%	278 (86.07%)	95.99%

150 meters	418 (61.74%)	623 (38.34%)	TWM	313 (50.24%)	48.47%	398 (63.88%)	61.85%	461 (74.00%)	72.46%
			SWM	292 (46.87%)	61.65%	375 (60.19%)	73.47%	440 (70.63%)	83.23%

200 meters	527 (77.84%)	908 (55.88%)	TWM	446 (49.12%)	47.16%	568 (62.56%)	60.57%	644 (70.93%)	70.01%
			SWM	418 (46.04%)	61.09%	522 (57.49%)	71.83%	618 (68.06%)	80.20%

250 meters	595 (87.89%)	1117 (68.74%)	TWM	584 (52.28%)	50.81%	723 (64.73%)	63.15%	818 (73.23%)	72.58%
			SWM	549 (49.15%)	61.04%	674 (60.34%)	74.43%	781 (69.92%)	80.6%

300 meters	623 (92.02%)	1271 (78.22%)	TWM	704 (55.39%)	53.88%	861 (67.74%)	66.42%	999 (78.60%)	77.43%
			SWM	658 (51.77%)	66.25%	827 (65.07%)	76.01%	951 (74.82%)	82.57%

350 meters	646 (95.57%)	1380 (84.92%)	TWM	834 (60.43%)	59.27%	1014 (73.48%)	72.10%	1125 (81.52%)	81.04%
			SWM	804 (58.26%)	69.58%	969 (70.22%)	80.85%	1098 (79.57%)	86.17%

400 meters	657 (97.05%)	1440 (88.62%)	TWM	920 (63.89%)	63.23%	1107 (76.88%)	76.20%	1261 (87.57%)	86.89%
			SWM	880 (61.11%)	74.81%	1068 (74.17%)	83.67%	1204 (83.61%)	89.55%

450 meters	660 (97.49%)	1473 (90.65%)	TWM	1037 (70.40%)	69.32%	1227 (83.30%)	82.40%	1337 (90.77%)	90.28%
			SWM	989 (67.14%)	76.37%	1184 (80.38%)	87.07%	1300 (88.26%)	92.22%

500 meters	664 (98.08%)	1501 (92.37%)	TWM	1148 (76.48%)	75.26%	1307 (87.08%)	86.61%	1400 (93.27%)	92.72%
			SWM	1126 (75.02%)	81.00%	1277 (85.08%)	89.15%	1376 (91.67%)	94.83%

**Table 3:** Comparison of selected 7-Eleven stores at different population densities and for different neighborhood types.

	TWM		SWM		TWM-SWM overlap	
	100 meters	300 meters	100 meters	300 meters	100 meters	300 meters
(a) Population density						
Low	30	22	31	30	13	11
Middle	30	39	29	28	15	16
High	40	39	40	42	23	18
Total	**100**	**100**	**100**	**100**	**51**	**45**
(b) Neighborhood type						
Commercial	31	29	27	22	14	11
Residential	47	54	53	62	25	28
Other	22	17	20	16	12	6
Total	**100**	**100**	**100**	**100**	**51**	**45**

**Table 4:** Average and standard deviation for distances from each 7-Eleven store to the nearest fire station (meters).

Weighting scheme and statistics: min, max, mean, SD		AED service range			
		100 meters		300 meters	
		Mean	SD	Mean	SD
TWM	4.68, 34.46, 9.56, 1.67	869	429	881	458
SWM	12.39, 39796162.90, 1198850.25, 1801599.73	1,230	344	1,303	432
